# GOMCL: a toolkit to cluster, evaluate, and extract non-redundant associations of Gene Ontology-based functions

**DOI:** 10.1186/s12859-020-3447-4

**Published:** 2020-04-10

**Authors:** Guannan Wang, Dong-Ha Oh, Maheshi Dassanayake

**Affiliations:** Department of Biological Sciences, Louisiana State University, Baton Rouge, LA USA

**Keywords:** Gene ontology clustering, GO annotations, Functional networks, GO similarity, Markov clustering, Functional genomics, High throughput omics

## Abstract

**Background:**

Functional enrichment of genes and pathways based on Gene Ontology (GO) has been widely used to describe the results of various -omics analyses. GO terms statistically overrepresented within a set of a large number of genes are typically used to describe the main functional attributes of the gene set. However, these lists of overrepresented GO terms are often too large and contains redundant overlapping GO terms hindering informative functional interpretations.

**Results:**

We developed GOMCL to reduce redundancy and summarize lists of GO terms effectively and informatively. This lightweight python toolkit efficiently identifies clusters within a list of GO terms using the Markov Clustering (MCL) algorithm, based on the overlap of gene members between GO terms. GOMCL facilitates biological interpretation of a large number of GO terms by condensing them into GO clusters representing non-overlapping functional themes. It enables visualizing GO clusters as a heatmap, networks based on either overlap of members or hierarchy among GO terms, and tables with depth and cluster information for each GO term. Each GO cluster generated by GOMCL can be evaluated and further divided into non-overlapping sub-clusters using the GOMCL-sub module. The outputs from both GOMCL and GOMCL-sub can be imported to Cytoscape for additional visualization effects.

**Conclusions:**

GOMCL is a convenient toolkit to cluster, evaluate, and extract non-redundant associations of Gene Ontology-based functions. GOMCL helps researchers to reduce time spent on manual curation of large lists of GO terms, minimize biases introduced by redundant GO terms in data interpretation, and batch processing of multiple GO enrichment datasets. A user guide, a test dataset, and the source code of GOMCL are available at https://github.com/Guannan-Wang/GOMCL and www.lsugenomics.org.

## Background

High-throughput “omics” approaches are frequently employed to investigate expression changes and regulation of genes at a genome-wide level. Use of these genomic data often results in the identification of large lists of genes of interest. A standard approach to summarize the functions of these genes is to determine the enriched functions represented by Gene Ontology (GO) terms and other functional associations extracted from databases such as KEGG [1–3], Reactome [4] and Pathway Commons [5], known as pathway enrichment analysis [6, 7]. This approach significantly simplifies the need from understanding the biological meaning embedded in individual genes in a large list, to the interpretation of enriched gene sets that could serve as a summary of enriched functions.

GO resources have become the most widely used knowledge base in terms of gene functions [8, 9], which provides a controlled hierarchy of GO vocabularies describing biological processes, molecular functions, and cellular components. However, this hierarchical functional annotation system presents a high level of redundancy as parent GO terms include or partially overlap with child GO terms and one gene could be annotated with seemingly unrelated GO terms. The computational tool, Enrichment Map [10, 11] was initially developed to overcome this problem by building a GO similarity network built on the overlap between gene sets annotated with each GO term. Yet, the identification of GO clusters within the GO similarity network in Enrichment Map does not define clusters and therefore the user has to separate groups based on a visual selection, which can be heavily affected by the layout of the network visualizations. As a result, when there are large numbers of similar GO terms, it is challenging to identify significant functional groups using Enrichment Map. Another comparable tool, ClueGO, identifies functional groups by first creating all possible initial groups with a user-defined number of GO terms showing similarities equal or above the predefined threshold and then iteratively comparing and merging these initial groups if the overlap between them is above the predefined threshold [12]. However, ClueGO can assign unique GO terms to multiple groups, making it challenging to identify non-redundant clusters [12]. Additionally, this tool does not accept direct output files from other commonly used GO enrichment tools such as BiNGO, g:Profiler, or agriGO. Both tools fall short at parallel processing a large number of distinct set of gene functions often encountered in large-scale -omics experiments. To address these limitations and generate a similarity-based functional GO network, we developed a new toolkit, GOMCL, that applies the Markov Clustering (MCL) algorithm [13–15] to identify cluster structures in GO networks in an unbiased approach. Each GO term is represented by a node and edges connect two GO terms that share a certain percentage of gene members in GOMCL. To further facilitate the interpretation of resulting functional groups, GOMCL allows users to generate hierarchy plots and provides sub-clustering options for any number of selected clusters. GOMCL is a user friendly python toolkit, which offers multiple visualization schemes and enables batch processing of large GO datasets to mine for functionally significant attributes.

### Implementation

**Fig. 1:**
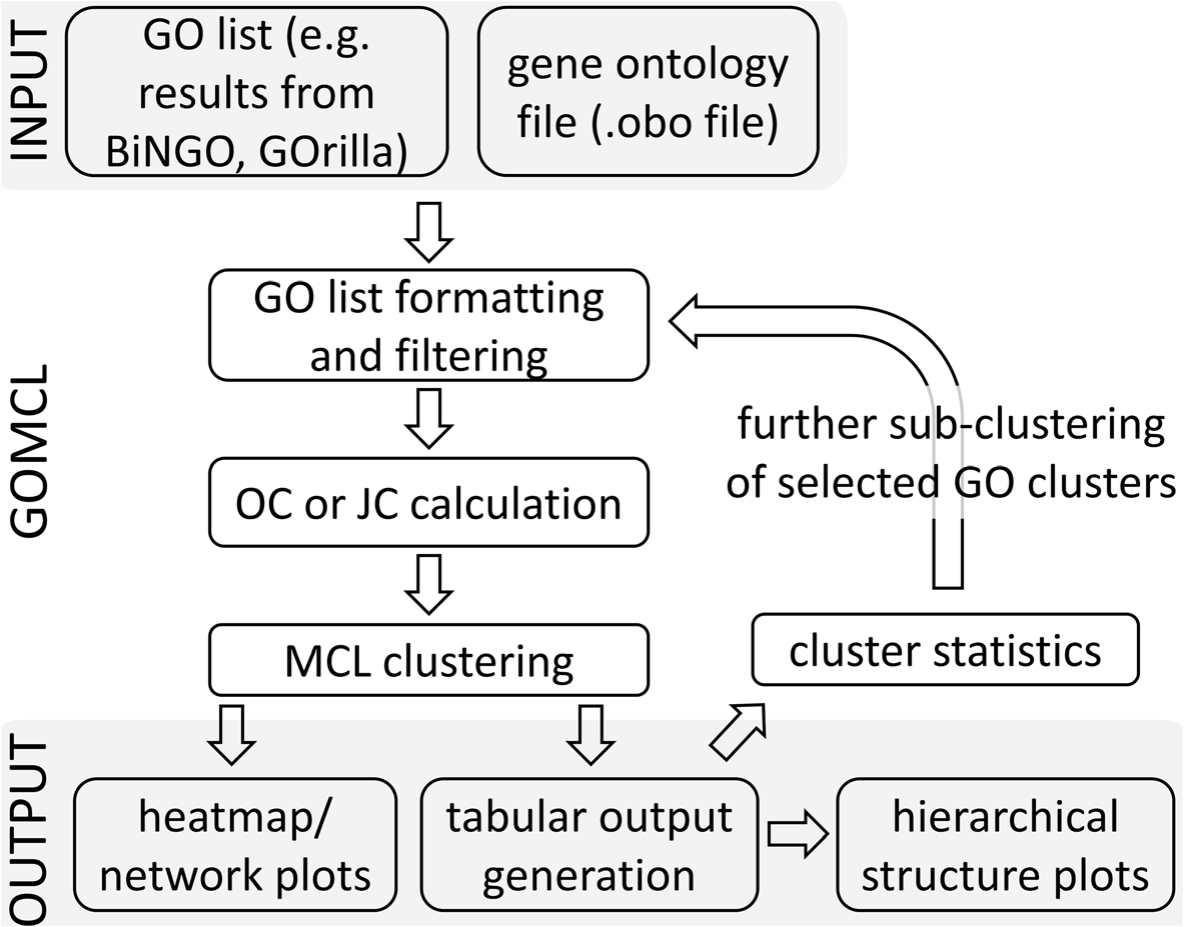
The workflow of GOMCL clustering on GO enrichment test results

GOMCL is implemented in Python and allows grouping of lists of individual GO terms of interest into GO clusters using MCL (Fig. 1). GOMCL encapsulates its entire pipeline in a single command and offers default parameters with which users can expect optimal results.

### Input data

The package accepts the direct outputs from a variety of commonly used GO enrichment analysis tools, including BiNGO [16], GOrilla [17], g:Profiler [18], and agriGO [19], as well as customized GO lists. Support for more enrichment tools will be provided. In addition to the GO lists, GOMCL requires a GO ontology file in OBO format from the Gene Ontology Consortium (http://geneontology.org/) as an input (Fig. 1).

### GOMCL workflow

GOMCL first trims the input GO lists by removing overly broad GO terms whose size is greater than a user-defined threshold. For example, a large GO term such as biological regulation (GO:0065007) has over 12,000 child GO terms, including 15,000 genes in Arabidopsis and is often uninformative as a term representing a meaningful biological function. GOMCL also enables users to separate input GO lists into biological process, molecular function, and cellular component categories or any combinations of these categories if clustering within different categories is preferred. Each term in the trimmed GO lists is then compared to each other, and similarity between any two GO terms is computed based on the overlaps between the members of these two GO terms as either a *Jaccard Coefficient* (*JC*) or an *Overlap Coefficient* (*OC*) [10]. Given any two GO terms, A and B, the *Jaccard Coefficient* (*JC*) is calculated as A∩B/A∪B, and preferred for clustering of similarly sized GO terms. The *Overlap Coefficient* (*OC*) is derived from A∩B/min (A, B), and works better to maximally reduce the redundancy between disproportionately sized GO terms. The construction of the GO term similarity network is initiated using only those interactions that pass a user-defined threshold for the *Jaccard* or *Overlap coefficient* of users’ choice. MCL algorithm is subsequently applied to identify cluster structure in the initial network and assigns more similar GO terms into one cluster. The resulting GO clusters are ordered based on the number of genes in each cluster. GO terms with largest number of genes, or smallest enrichment *p*-value, or most other GO terms connected are selected and offered as potential representative GO terms for each cluster. GOMCL also reproduces the hierarchy of GO terms from the provided ontology structure for any user-selected clusters upon command to assist identification and interpretation of the functional themes of these clusters. A novel functionality enabled in GOMCL that is unavailable in previous tools for GO-network analysis, is the evaluation of clustering results by visualizing the distribution of similarity indexes between GO terms for each cluster. Taking this one step further, GOMCL includes a second module, called GOMCL-sub, which provides users customizable options to break down selected clusters produced by GOMCL into sub-groups with more specific functional themes. These functionalities combined, allows users to determine if there are distinct functional themes present in primary clusters and further identify these sub-structures in clusters of interest.

### Output format

**Fig. 2:**
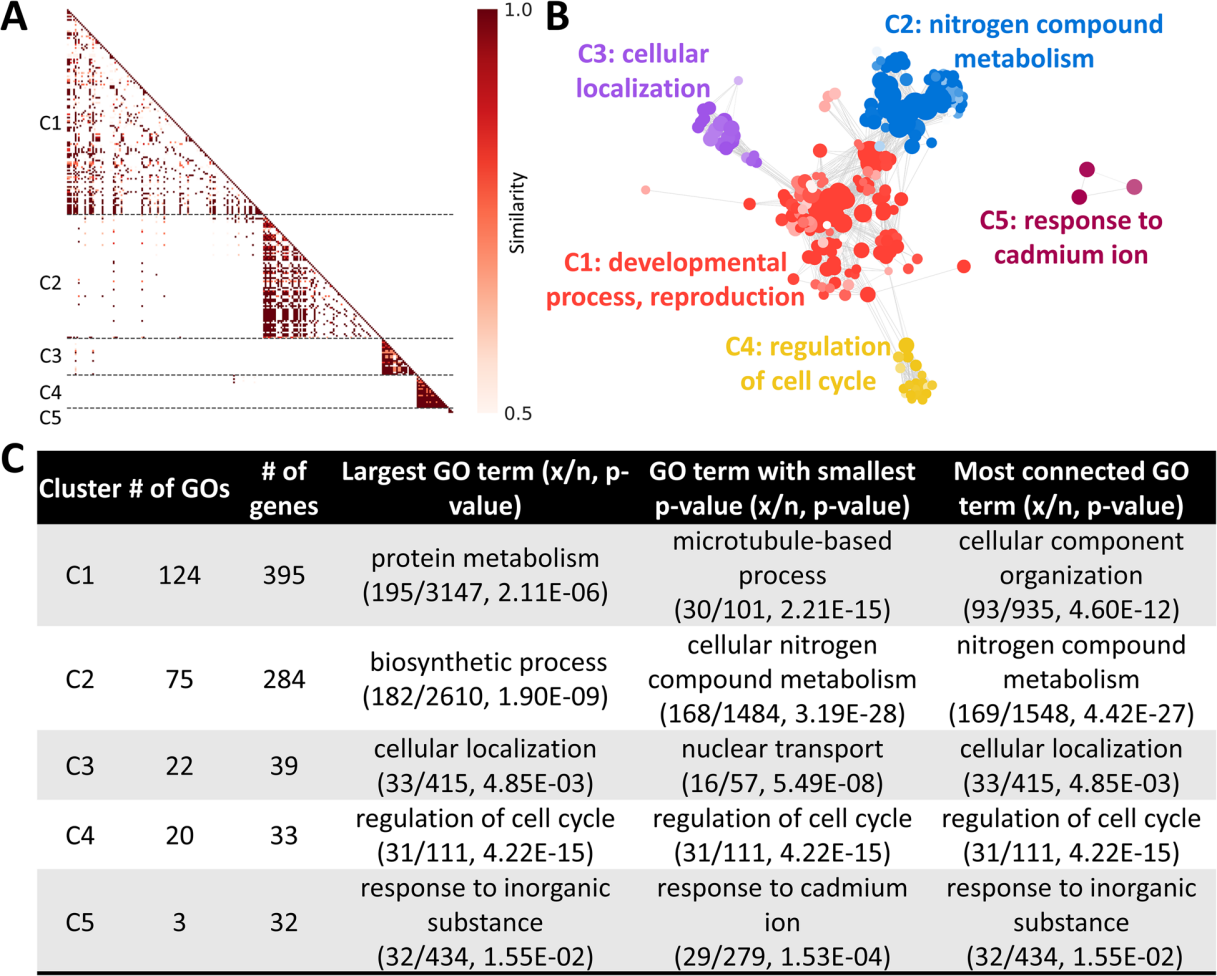
Representative outputs created with GOMCL for clustering of enriched GO terms in a selected study [22] to distinguish two cell populations. Overlap coefficient of 0.5 and cluster granularity of 1.5 were used in GOMCL for cluster identification. **a** Similarity heatmap, **b** Network of identified GOMCL clusters. Node size represents the number of genes in the test set which are annotated to that GO term; edges represent the similarity index between GO terms; each cluster is coded with a different color; and shade of each node represents *p*-value assigned by the enrichment test. Lighter to darker shades indicate larger to smaller *p*-values, respectively. **c** A tabular summary of all GOMCL clusters. x: the number of genes in the test set; n: total number of genes in the reference annotation

**Fig. 3:**
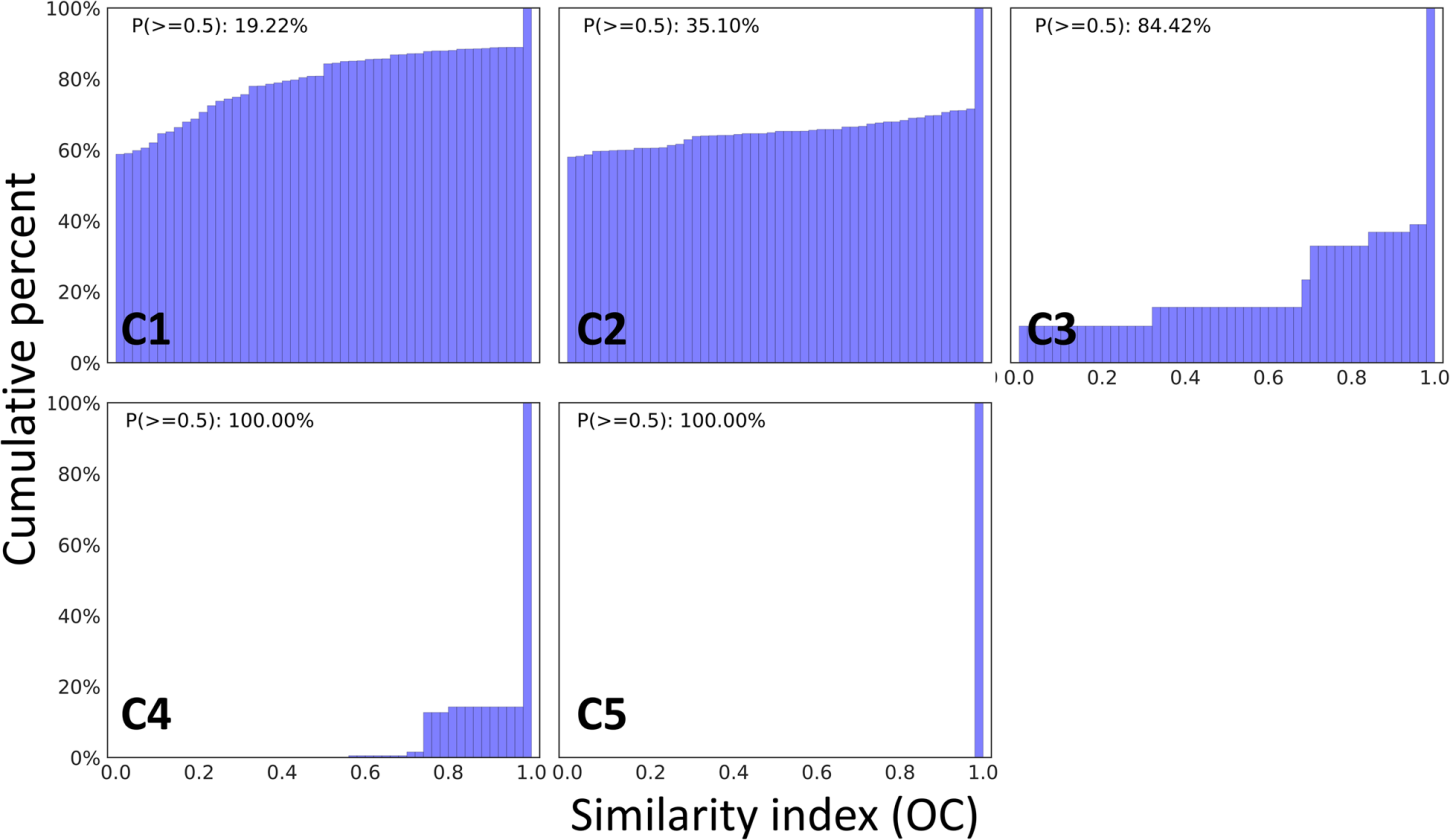
Cumulative distribution of similarity indexes between GO terms within each GOMCL cluster identified from test data reported in Wendrich et al. 2017. *P*(> = 0.5) indicates the proportion of similarity indexes greater than 0.5 among all the similarity indexes within a given cluster

**Fig. 4:**
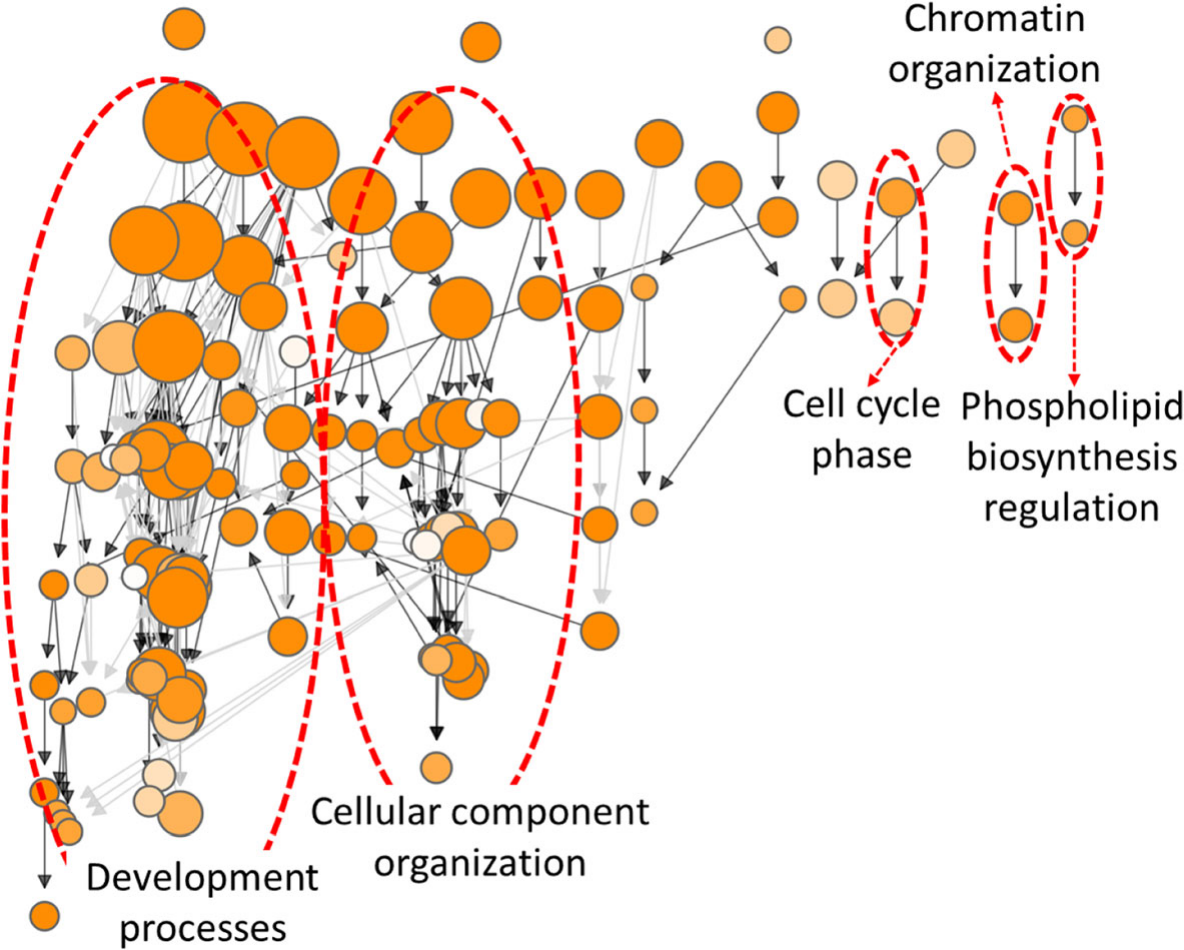
GO hierarchical structure produced using GOMCL for cluster C1 described in Fig. 1. Edges represent the parent/child relationships of the GO terms. The black edges connect parent and child terms that are directly linked, while the grey edges indicate connections with intermediate GO terms between the parent and child terms. Node size represents the number of genes in the test set which are annotated to that GO term; and shade of each node represents *p*-value assigned by the enrichment test. Lighter to darker shades indicate larger to smaller *p*-values, respectively. The main hierarchical branches are marked by red circles

The standard GOMCL output consists of a heatmap (Fig. 2a), a graphical GO-similarity-network based on the clustering results (Fig. 2b), a tabulation of each GO term with cluster information and depth information [20] from the provided ontology structure, and a summary file for all clusters (Fig. 2c). Graphical presentations of similarity index distribution (Fig. 3) and GO hierarchy for individual clusters (Fig. 4) are generated if the user chooses that option to create additional result files. If the user plans to generate cluster depth information for each GO term and build GO hierarchies as an output file, we recommend that the same version of the GO ontology file used in the GO enrichment analysis tool where the GO input list is created to should be used as an input for GOMCL. In addition to the graphical outputs, the user can opt to generate simple interaction format files with either similarity between GO terms or GO hierarchy (Additional file 2), both of which can be directly used as inputs, together with the information about clustered GO terms (Additional file 1), to Cytoscape [21] for further manipulation of GO network visualization.

## Results

As a proof of concept, we performed a GOMCL run on a list of over-represented GO terms identified from genes differentially expressed between two GFP tagged cell populations of Arabidopsis roots in a published study [22] to highlight the functional use of GOMCL. In this published study, a xylem-specific promoter was used to drive the expression of GFP in Arabidopsis, and root proximal meristem cells were later separated into two populations based on the intensity of GFP signals. Cells with high GFP signals were assumed to be close to the quiescent center while cells with low GFP signals were assumed to be located away from the quiescent center. A microarray analysis was then conducted to compare the two cell populations and the authors aimed to see a difference in gene expression associated with cell division between these two cell populations.

### Cluster identification

We used GO terms that had less than 3500 genes annotated under each GO annotation for Arabidopsis, to allow identification of specific functional traits associated with the published study. This resulted in 244 total GO terms (out of 251) enriched in genes expressed higher in the cell population with high GFP intensity (Additional file 1). The default *Overlap Coefficient* of 0.5 and granularity of 1.5 were used for cluster identification. These cutoffs can be set by the user. Among the 244 GO terms, GOMCL identified five distinct clusters with minimal overlap between clusters and extensive overlaps among GO terms within each cluster (Fig. 2a). The largest cluster (C1) included 124 GO terms and was mainly related to developmental processes and reproduction (Fig. 2b, c). The 4th largest cluster, albeit comprising only 33 genes from 20 GO terms (Fig. 2b, c), was overrepresented in genes associated with regulation of cell cycle and was also found to be mostly associated with the largest cluster. These representative functional groups and associations reflected the essential difference in the growth stages between the high GFP cells (cells assumed to be close to the quiescent center) and low GFP cells (cells located away from the quiescent center) in the targeted study where it aimed to see a difference in gene expression associated with cell division between these two cell populations [22]. The reduction from over 200 GO terms to 5 GO clusters preserved the enriched functional themes and facilitated the explanation of major patterns identified among ~ 1000 differentially expressed genes.

### Cluster quality evaluation and sub-clustering process

**Fig. 5:**
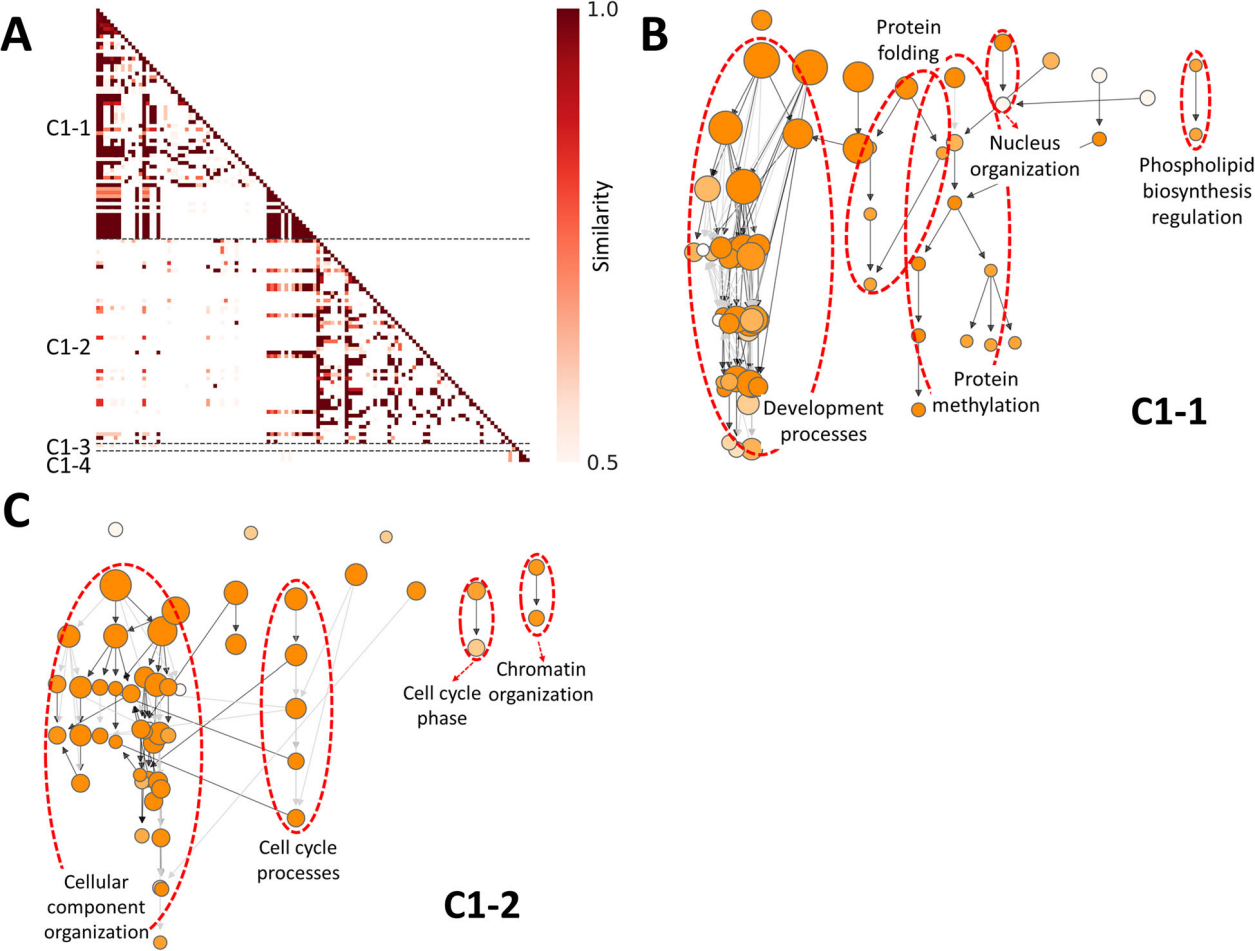
Sub-clustering results produced by GOMCL-sub on cluster C1 described in Fig. 1. **a** Similarity heatmap of sub-groups identified by GOMCL-sub. **b** and **c** GO hierarchical structures of C1–1 and C1–2 sub-clusters. The black edges connect parent and child terms that are directly linked, while the grey edges indicate connections with intermediate GO terms between the parent and child terms. Node size represents the number of genes in the test set which are annotated to that GO term; and shade of each node represents *p*-value assigned by the enrichment test. Lighter to darker shades indicate larger to smaller *p*-values, respectively. The main hierarchical branches are marked by red circles

To demonstrate the use of cluster quality evaluation and sub-clustering, we first enabled options to generate similarity index distributions for all five clusters identified by GOMCL. As shown in Fig. 3, the majority of similarity indexes between GO terms within cluster C3, C4, and C5 were greater than 0.5. However, there is a large proportion of GO terms from cluster C1 and C2 showing no or small overlaps with other GO terms from the same cluster. To assist determining whether cluster C1 and C2 should be further separated into groups with more specific functional themes, we plotted GO hierarchies for these two clusters with GOMCL parameter -hg on (Fig. 4, Additional file 3). Several distinct branches were identified from the hierarchical structures of GO terms within each cluster (Fig. 4, Additional file 3), indicating the possible presence of distinct sub-groups within these clusters. We further employed GOMCL-sub, which was designed to analyze selected clusters produced by GOMCL, to identify the sub-groups within large clusters such as C1 and C2 when users need to identify more distinct and functionally informative sub-clusters. To increase clustering sensitivity, we reduced the cutoff of GO term size to 2000 and increased the granularity to 1.8, and left similarity cutoff unchanged. With these parameters, GOMCL-sub passed 122 GO terms from cluster C1 and was able to separate them into 4 sub-groups (Fig. 5a). 117 out of the 122 GO terms were assigned to the two largest sub-groups (C1–1 and C1–2), whose main functional themes were associated with development processes and cellular component organization, respectively (Additional file 4). These two sub-groups recapitulated and extended the main theme of the original cluster, C1. Additionally, this led to the identification of more informative details of the cluster. For instance, the development process associated with cluster C1–1 was mainly composed of GO terms involved in anatomical development processes (e.g. root development) (Fig. 5b, Additional file 5). Whereas, over 85% of the GO terms in cluster C1–2 were involved in processes such as cellular component organization that represented chromosome organization and cytoskeleton organization. More importantly, cell cycle processes were found to intersect with cellular component organization and seemed to link different processes assigned to the C1–2 sub cluster (Fig. 5c, Additional file 5). These results combined, provided a more detailed overview of how development processes and cell cycle regulation were different between the two cell populations in the target study. Contrasting to cluster C1, cluster C2 did not appear to contain additional sub-cluster structure that could be separated further, when GOMCL-sub was applied. The visualization of GO hierarchy of GOMCL clusters and further identification of sub-groups with more specific functional themes by GOMCL-sub greatly contribute to biological interpretations by facilitating objective clustering and extraction of overrepresented functional associations.

Notably, GOMCL identified these 5 clusters out of a list of 251 GO terms and generated associated result files in ~ 2 min while using only 300 Mb of RAM (easily found in most desktops/laptops). GOMCL-sub further separated cluster C1 and C2 clusters in less than 2 mins with similar amount of memory used. Given the efficiency of the toolkit, this can be easily implemented to conduct batch processing of multiple datasets associated with large –omics datasets.

While the proof of concept analyses described above using GOMCL is used to highlight functional associations drawn from a typical RNAseq experiment, the use of GOMCL is not limited to summarizing functional processes from RNAseq data. For example, it has been recently successfully used in summarizing gene functions associated with multiple epigenetic marks in rice under phosphorus starved conditions [23]. Additionally, GOMCL can be used to cluster and summarize biological processes associated with GO-slim ontologies [24], similar to its use with the standard GO terms. To further assess and highlight its versatility, we compared the features of GOMCL to several existing GO term clustering tools in Table 1.

**Table 1:** Feature comparison of GOMCL and other tools for clustering of GO terms

	GOMCL	Enrichment Map [10]	ClueGO [12]	DAVID [25]	POSOC [26]
Clustering	Yes	Yes	Yes	Yes	Yes
Clustering basis	*Jaccard Coefficient*/*Overlap Coefficient*	*Jaccard Coefficient*/*Overlap Coefficient*	Kappa statistics	Kappa statistics	GO hierarchical distance
Clustering method	Markov Clustering (MCL) algorithm	Visual identification	Iterative merging	Iterative merging	Ranking
Sub-clustering	Yes	No	No	No	No
Type of visualization support	Network and Hierarchy	Network	Network	Table	Table
Compatible with other enrichment tools	Yes	Yes	No	No	No
Batch processing	Yes	No	No	No	No

## Discussion

GOMCL is an open-source Python toolkit to identify clusters among GO term similarity networks using the MCL clustering algorithm. This toolkit allows grouping of GO terms into functional clusters to further simplify the interpretation of large datasets, reduce redundancy of functional interpretations, and especially when visual identification of cluster structure is not feasible due to a large number of enriched GO terms often found in -omics data (see Table 1 for a comparison with other available tools). To better evaluate and understand the resulting clusters, GOMCL offers options to visualize similarity indexes between GO terms and GO hierarchy. A second module, GOMCL-sub, is further introduced to further examine large clusters when users suspect that two or more distinct minimally overlapping functions might be captured in one large cluster. We demonstrated the use of GOMCL in successfully capturing the functional themes associated with a published study [22] as proof of concept of the toolkit. We showed that GOMCL built a concise and informative view of biological processes different between the two conditions tested in the target study and summarized the main differences. It was also demonstrated that sub-clustering enabled by GOMCL-sub was able to provide additional insight of selected clusters produced by GOMCL to guide further investigation.

GOMCL can be used for batch processing of multiple enrichment test results defined by the user. It is applicable for any research project where lists of genes of interest are generated. It is compatible with a wide variety of GO enrichment analysis tools publicly available, which would reduce intermediate steps needed to convert different input formats to conform to GOMCL requirements.

GOMCL currently uses the MCL algorithm for cluster identification and is compatible with commonly-used GO enrichment tools. For future versions, we consider implementing additional clustering methods, improving labeling of nodes, and supporting more GO enrichment analysis tools.

## Conclusion

Lists of overrepresented GO functions from GO enrichment analyses are often long and redundant. We present GOMCL as a convenient toolkit to identify functional clusters among GO term similarity networks and further separate the resulting clusters into more informative sub-groups. It enables the user to effectively summarize long lists of GO functions into biologically informative non-redundant clusters without hand-picked selections and look for major functional themes associated with the experiments. GOMCL assists with the unmet yet increasing need for interpreting large gene sets often produced from -omics studies.

## Supplementary information

**Additional file 1.** GOMCL cluster information for each GO term.

**Additional file 2.** Similarity connections of GOMCL clusters and hierarchical connections of GOMCL cluster C1.

**Additional file 3.** GO hierarchical structure produced using GOMCL for cluster C2 described in Fig. 1.

**Additional file 4.** Summary for GOMCL-sub clustering results of C1 described in Fig. 1.

**Additional file 5.** GOMCL-sub sub-cluster information for each GO term in cluster C1 described in Fig. 1.

## Data Availability

The source code of GOMCL is freely available at https://github.com/Guannan-Wang/GOMCL and www.lsugenomics.org. The code is provided with a detailed manual and a sample test dataset used in the current study. Installation guidelines for GOMCL and associated dependencies and detailed explanations for parameters in GOMCL are available on the GitHub page.

## Abbreviations

GO: Gene Ontology
MCL: Markov Clustering
JC: Jaccard Coefficient
OC: Overlap Coefficient
