# Supplementary material for: GOMCL: a toolkit to cluster, evaluate, and extract non-redundant associations of Gene Ontology-based functions
Source: BMC Bioinformatics. 2020 Apr 10;21:139. doi: 10.1186/s12859-020-3447-4 (PMC7146957; doi:10.1186/s12859-020-3447-4)
Supplement: Supplementary file 3 — Additional file 3. GO hierarchical structure produced using GOMCL for cluster C2 described in Fig. 1. [file 12859_2020_3447_MOESM3_ESM.docx]

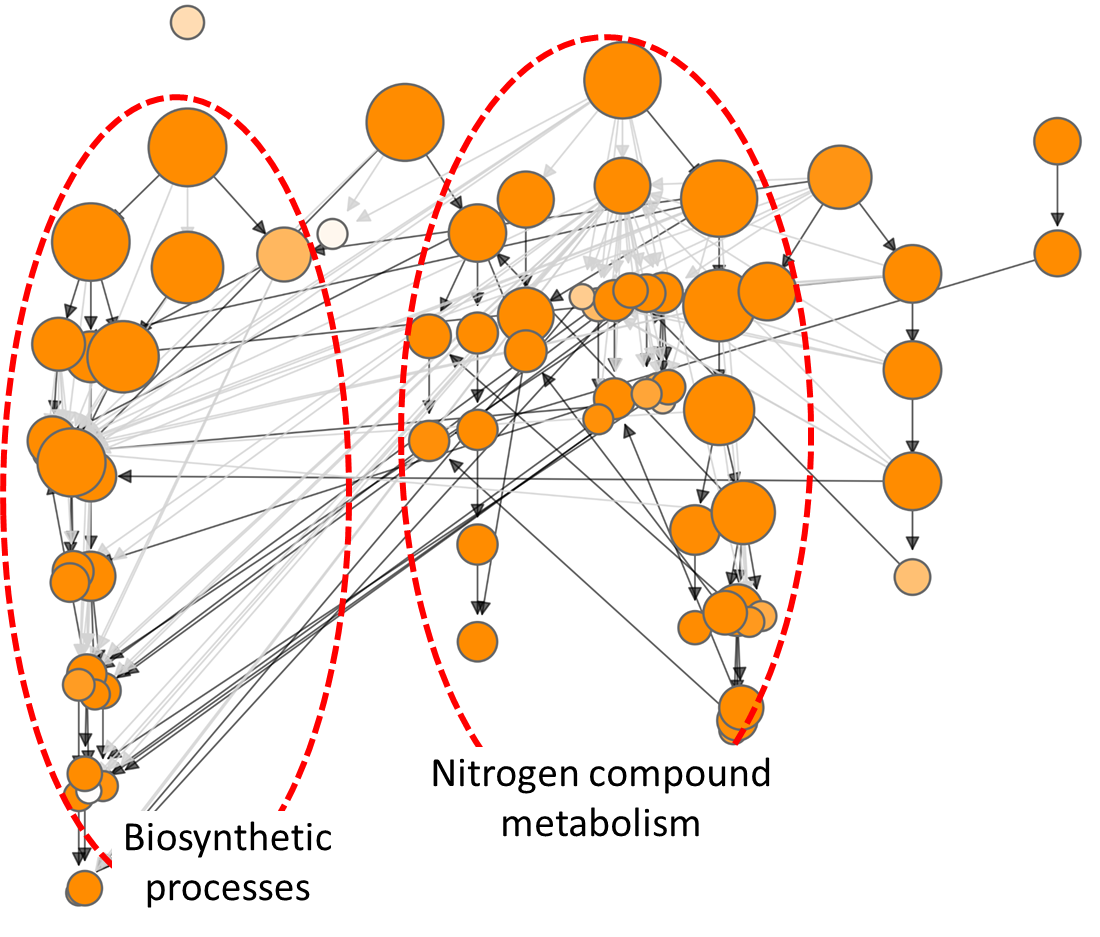


Figure S1. GO hierarchical structure produced using GOMCL for cluster C2 described in Figure 1. Edges represent the parent/child relationships of the GO terms. The black edges connect parent and child terms that are directly linked, while the grey edges indicate connections with intermediate GO terms between the parent and child terms. Node size represents the number of genes in the test set which are annotated to that GO term; and shade of each node represents p-value assigned by the enrichment test. Lighter to darker shades indicate larger to smaller p-values, respectively. The main hierarchical branches are marked by red circles.
